# Essential pediatric hypertension: defining the educational needs of primary care pediatricians

**DOI:** 10.1186/1472-6920-14-154

**Published:** 2014-07-27

**Authors:** Stephen D Cha, Deena J Chisolm, John D Mahan

**Affiliations:** 1 Pediatric Nephrology, Nationwide Children’s Hospital, Columbus, OH, USA; 2 The Ohio State University College of Medicine, Columbus, OH, USA; 3 The Research Institute at Nationwide Children’s Hospital, Columbus, OH, USA

**Keywords:** Pediatric hypertension, Primary care pediatricians, Primary care physicians, Medical education

## Abstract

**Background:**

In order to better understand the educational needs regarding appropriate recognition, diagnosis and management of pediatric hypertension (HTN), we asked practicing pediatricians questions regarding their educational needs and comfort level on this topic.

**Methods:**

We conducted 4 focus group sessions that included 27 participants representing pediatric residents, adolescent medicine physicians, clinic based pediatricians and office based pediatricians. Each focus group session lasted for approximately an hour and 90 pages of total transcriptions were produced verbatim from audio recordings.

**Results:**

Four reviewers read each transcript and themes were elucidated from these transcripts. Overall, 5 major themes related to educational needs and clinical concerns were found: utilization of resources to define blood pressure (BP), correct BP measurement method(s), co-morbidities, barriers to care, and experience level with HTN. Six minor themes were also identified: differences in BP measurement, accuracy of BP, recognition of HTN, practice pattern of care, education of families and patients, and differences in level of training. The focus group participants were also questioned on their preferences regarding educational methods (i.e. e-learning, small group sessions, self-study, large group presentations) and revealed varied teaching and learning preferences.

**Conclusions:**

There are multiple methods to approach education regarding pediatric HTN for primary care pediatricians based on provider preferences and multiple educational activities should be pursued to achieve best outcomes. Based on this data, the next direction will be to develop and deliver multiple educational methods and to evaluate the impact on practice patterns of care for children and adolescents with HTN.

## Background

HTN is becoming a more commonly encountered medical condition in the pediatric population [1-3]. In the past, pediatric HTN was thought to be mostly due to a secondary cause that could be identified and treated. However, this thinking has changed in the past several decades and it has become more apparent that primary, or essential HTN, is not encountered only in adults but is becoming more prevalent in pediatrics [2,4-6]. This observation has mirrored the obesity epidemic in children, adolescents and even pre-teen children [3,5-11].

In childhood, HTN is often not a silent disease but rather many children will have issues with headaches, difficulties with sleep initiation and daytime somnolence [12]. Elevated BP’s in childhood can have profound cardiovascular and other impacts in adulthood [1,13-18].

Since essential HTN is becoming more prevalent in pediatrics, it is incumbent on our community of pediatricians to become more vigilant in correctly recognizing and initiating appropriate evaluation of children with essential HTN [19,20]. In order to accomplish this goal, the parameters that constitute HTN in children and adolescents and the ability to obtain accurate and reliable blood pressure (BP) measurements must be well understood. The value of parsing out those patients with essential HTN who may respond to therapeutic lifestyle changes (TLC) from those with secondary HTN who will require correction or medical treatment will only be intensified as pediatric HTN becomes a bigger health problem. Regardless of cause, all hypertensive children and adolescents should benefit from implementation of TLC (i.e., healthy eating and exercise) [21]; a substantial subset will likely benefit from antihypertensive medications if HTN persists and/or is severe despite TLC.

We are interested in developing effective educational methods to reinforce proper recognition, diagnosis, and management of HTN in children and adolescents. In order to develop meaningful and beneficial educational methods, we first sought to ascertain the education needs and preferences of the local practicing pediatricians and pediatric residents in our community with the assumption that these findings can help inform and shape our future efforts and also inform others interested in addressing this educational need.

## Methods

### Study design

We utilized focus groups [22] to gain a better understanding of the educational needs regarding recognition, diagnosis and management of pediatric HTN amongst local pediatricians and pediatric residents practicing in Columbus, OH. Focus groups were selected as our method for eliciting needs in order to collect qualitative, unstructured responses and to capitalize on within-group discussions. Four focus groups were conducted between October 2011 and December 2011. Our local IRB determined that this project does not fit under the definition of human subjects research under 45 CFR part 46, or 21 CFR part 50 and therefore a formal IRB process was waived. Verbal consent for participation and recording was obtained from all of the participants. The focus group materials consisted of three pediatric HTN scenarios that were discussed with questions interspersed throughout the case discussion. The first case was a healthy 6 year old boy with elevated readings, the second case was an overweight 16 year old adolescent female and the third case was a 15 year old male athlete who had clear cut hypertension. The purpose of these cases was to present possible, real-life outpatient clinical scenarios in order to assess the participants’ baseline information regarding their practice behaviors and referral patterns. We also questioned our participants regarding their teaching and learning preferences. Open-ended questions were used to help guide the discussion and the moderator had limited influence during the focus group sessions [23,24]. Discussion amongst participants was encouraged with the goal of understanding knowledge deficits, areas of perceived competence and educational preferences.

### Focus group process

Invitations for participation were sent to recruit pediatricians via email. These participants included 2^nd^ and 3^rd^ year pediatric residents, adolescent medicine physicians and clinic based pediatricians employed by Nationwide Children’s Hospital (NCH) and a group of office based pediatricians affiliated with NCH. These groups are representative of the primary care pediatricians (PCP) who provide care for our pediatric population in our community. As incentive, coffee and food was offered during the session as a token of appreciation for participation. Each session lasted approximately 45–60 minutes. The sessions were moderated by at least one investigator and a co-investigator was present to assist in navigating the conversation at two of the sessions. Each focus group session was audio-recorded and transcribed verbatim to increase analytic accuracy.

### Population studied

There were 27 participants and each participant actively cared for pediatric patients and encountered pediatric HTN in her/his scope of practice. Each focus group had at minimum 6 participants. The sessions were held in a conference room at NCH in either the early morning or during a lunch hour. Six of the participants primarily saw adolescents (adolescent medicine physicians). Ten participants were pediatric residents who were training at NCH. The other 11 participants were comprised of 6 community pediatricians and 5 clinic pediatricians. Of the 27 participants, 10 were male and 17 were female.

### Analysis

Each session was transcribed verbatim to produce 90 pages of single spaced transcripts for analysis using thematic analysis [25-27]. Four reviewers read each transcript and identified various themes that emerged related to provider educational needs and clinical concerns [25,28]. Each reviewer found varied themes but after comparing all the themes, patterns were identified among the reviewers’ comments and from these comments, 5 major themes and 6 minor themes were elucidated. We present the themes and discuss the significance of our findings. Our methods and analysis were consistent with the RATS guideline for qualitative research [29].

## Results

Eleven themes emerged from the transcriptions regarding recognition, diagnosis and management of essential pediatric HTN. Of the 11, it became apparent that 5 themes were represented more often than the other 6. We divided the themes into major and minor categories.

### Major themes

Five themes were discussed more often than the rest and they were: utilization of resources to obtain BP, correct BP measurement method(s), co-morbidities, barriers to care, and experience level with HTN.

1. Correct BP measurement method(s): This theme centered around the issue of using manual versus automated methods to obtain BP readings. Most participants were in agreement that manual BP’s were more accurate. As a participant in one of the primary care pediatric groups said “But I would think that you would have to make sure that a manual (BP) matches the machine (BP) or something like that. Ours are all done manually”. This is an important issue for providers who recognize that if the BP measurement and value is inaccurate, then it cannot be used to accurately identify HTN in patients [30].

2. Utilization of decision support resources to recognize elevated BP: This theme centered around using available resources to correctly recognize and define BP in the real-time clinical setting. The Fourth Report Task Force tables containing BP values for boys and girls based on their height, gender and age was often mentioned. In some cases practitioners had these available through the electronic health record (EHR). A portion of the resident focus group transcription mentioned “Then I always use my curves…The percentiles, the ones in EPIC (NCH EHR)…That give me what percentile they are for gender, age”. Most of the other comments centered around the value of using BP percentile charts derived from the Fourth Report.

3. Co-morbidities: This theme dealt with the issue of other risk factors and co-morbid conditions that can be present in pediatric patients with HTN. The issues that emerged were those centered around healthy eating, dieting and obesity. One resident when referring to one of the scenarios said “I think…I am going to assume that her blood pressure is secondary to her obesity. I would say that sometimes that happens because someone’s overweight but obviously, there are possible health detrimental stuff because…and it might be affecting your health”. Supplement use was also raised as a concern as mentioned by a pediatrician “I would ask about supplements and a lot of the training things because a lot of the football players will take anything around. And I have seen a lot of kids who have elevated blood pressures who were on…um, I think it is called Spark…it has a whole bunch of caffeine in it”.[17].

4. Barriers to care: The issues that emerged in this theme centered around the barriers to care that exist for pediatric patients with HTN. A major concern identified was that the patients and families themselves could become a barrier to their own care. If the patients are not ready to address the problem, then the problem itself could not be remedied since a major part of treating HTN resolves around therapeutic lifestyle changes. As one resident mentioned “I guess assessing her readiness to change. If she doesn’t see it as a problem then it is going to be a bigger problem for you to get her to see that it is a problem”. Of note, family centered treatment was discussed by multiple participants. If the family members did not view the weight or BP as an issue, the participants noted less success with weight loss and change in eating habits [31].

5. Experience level with HTN: This theme was interesting in that each focus group varied in their comfort level based on their experiences regarding pediatric HTN. Within each focus group, there was also variability in comfort level based on personal experience. One resident mentioned that she was comfortable managing pediatric HTN but ended up referring the patient to outpatient nephrology clinic due to the lower comfort level of her clinic preceptor. “I think…my preceptors have not been traditionally super confidant managing it in the clinic setting and they would have probably have referred”. The converse was true for the preceptors who were comfortable managing HTN [32]. These practitioners often began the initial workup after appropriately recognizing HTN in their patients.

### Other themes

There were several other themes that also emerged. One pertained to differences in BP measurement related to clinical setting. This minor theme dealt with the issue of comparing BP’s taken in the inpatient versus outpatient setting. There was another theme about the accuracy of the BP reading. This theme dealt primarily with the state of the child during the BP measurement, with some participants recognizing that in some settings the child’s BP reading may be elevated due to another reason, (i.e., anxiety, fear), which raises the issue of whether specific readings are an accurate reflection of the patient’s “usual” BP. Another issue discussed was around the use of an appropriate sized cuff to obtain BP reading with participants noting that if too small of a cuff is used, then the patient’s BP reading may be falsely elevated, leading to an unnecessary work up in a normotensive child. Another theme centered around how the practitioner was notified of an elevated BP reading during their daily practice. Some practitioners had a “flag” that alerted them of an elevated BP reading in the patient’s EHR. This notification process was not uniform across the focus groups. More often, the practitioner has to recognize a potential hypertensive patient and know to look at the BP chart to determine if the reading is normal or elevated.

Practitioners amongst the focus groups and within each focus group had varied practice patterns, evaluation and treatment approaches to the child with HTN or suspected HTN. This also depended on the comfort level of each practitioner with pediatric HTN. Another theme dealt with the issue of educating the patient and family about the patient’s BP. Several participants noted that often the patient and/or family was not ready to be educated on the topic. However, other strategies emerged, such as methods to involve the family in the education process. One strategy employed by some of the FG participants involved connecting the patient’s BP to something that the patient was concerned about, such as their physical appearance. This strategy was employed more frequently in adolescents. Finally, different focus group participants were more comfortable managing a hypertensive pediatric patient than other participants. Some of this was due to the level of training received in medical school or residency but also determined by how often they saw hypertensive patients in their practice and the years of experience they had in dealing with pediatric HTN. Some of the practitioners who saw HTN more regularly stated that they felt comfortable managing essential HTN in children and adolescents.

### Learning preferences

The participants had varied responses regarding their preferences for learning material and settings for this topic. Overall, most agreed that a quick and easy-to-follow resource would be preferable. Most wanted a handout that recapitulated the major points regarding the approach to pediatric HTN. In terms of mastering the key content and skills, many also desired a reference that contained a flowsheet or algorithm for easy reference [33]. Most of the participants also stated that they preferred smaller group session presentations for learning with opportunities to review the key points in documents and/or online at a later date. Some practitioners preferred using only online learning methods and online resources. Most stated that they would use an online reference if one was easily assessable and not over-complicated [34].

## Discussion

As mentioned above, it was apparent that several themes emerged more frequently in regards to pediatric hypertension recognition, diagnosis and evaluation during these sessions than others (major themes or issues: utilization of resources to define BP, BP measurement method, co-morbidities, barriers to care, and experience level with HTN). Each theme provided different insights into the educational barriers and gaps regarding pediatric HTN for primary care practitioners. Of note, the major themes were varied in their content but each appeared important to many participants in specific ways. Two of the major themes stood out and deserve special attention: utilization of resources to obtain BP and BP measurement method. These 2 major themes were the most frequently mentioned and appeared to be straightforward opportunities to provide educational BP programs.

Utilization of decision support resources to recognize elevated BP is an important issue in that it addresses the challenge of accurately recognizing normal BP from elevated BP in pediatric patients of various sizes in the real-time clinical setting. The significance of this is derived from the fact that normal ranges for pediatric patients are defined by gender, age and height, a framework well recognized by all participants. This is important for the practitioner in real time since what may be considered HTN in one age group or height percentile may be normal for another age group or height percentile. For example, if two pediatric patients ten years apart have the same BP value, one value may be in the hypertensive range for one patient while being normal or even low for the older pediatric patient. Therefore, real time access to the pediatric BP charts (Fourth Report) needs to be readily available to practitioners in order to accurately appreciate the significance of each individual BP value. While using EHR to define BP in ranges and incorporate automatic notification of practitioners holds great promise to address this barrier, these are not yet readily available in all clinical settings.

A second important theme related to correct BP measurement method(s) as a key point of emphasis for the participants. If the BP isn’t taken correctly, practitioners realize that this value will not necessarily be reflective of the patient’s actual BP. For example, if the BP is not taken with the appropriate sized cuff in the right arm of a calm patient, the reading cannot be compared to the standard BP charts and is therefore not a useful measurement. The practitioners were uniformly aware of these issues but related concerns about how consistently other members of the health care team attended to these details. The “noise” conveyed from BP measurements in non-standardized settings could conflict with the standard techniques and records in the chart and they expressed desire for more standardized diagnostic and even treatment recommendations. More effective efforts to ensure proper BP measurement in clinical settings were identified as important priorities to accurately recognize HTN in children and adolescents. This theme also underscores opportunities to improve the care of children and adolescents in this area by implementing effective educational methods with all members of the pediatric health care team.

Understanding the 3 other major themes (co-morbidities, barriers to care, experience level with HTN) will also be important in development of educational methods.

1. Co-morbidities: Given the incidence of co-morbidities in children with hypertension, it will be important to address this issue with proper education and recognition of these co-morbidities (e.g., obesity, supplement use).

2. Barriers to care: In designing educational models for PCP, it is important to recognize the various barriers that PCP face when treating their hypertensive patients. Potential barriers to care in the patient population would include access to home BP equipment, access to medication, limited healthy food options, limited exercise opportunities and these barriers may impact our ability to provide optimal patient care for these patients.

3. Experience level with HTN: It is important that any education program builds on the variability of the PCP educational experiences to provide the necessary information and resources to enable the PCP to deliver the best care possible.

### Limitations

Several limitations need to be mentioned regarding this focus group study. First, several biases may exist in our study.

1. This study was conducted in a single institution so the results may have been biased based on the practice patterns of the physicians associated with our institution. Therefore our results may not be representative of other institutions.

2. This study was performed in an academic institution so the attitude and experiences amongst our practitioners may differ from those in a non-academic setting. Also, resource allocation may be very different between academic and non-academic institutions.

3. This study was conducted in NCH which is located in central Ohio and our practice patterns, values and patient population may be influenced by biases that pertain to central Ohio. The type of physicians who come to practice in this area of the country may have shared characteristics that may have influenced the results. Also, the patient demographics may be particular to the part of the country in a manner that may skew co-morbidities and prevalence of essential pediatric HTN.

4. This study was performed in a free standing children’s hospital and our practitioners have limited interaction with adult counterparts. If this study had been conducted in an adult hospital with a pediatric ward, the results may have been different due to the possibility of influence from adult colleagues.

A second limitation is that there was one moderator at 2 sessions and 2 moderators at the other 2 sessions. Since focus group sessions may not have been moderated in the same manner this may have introduced some variability in the results. For example, the discussion may have been led differently when only one moderator was present. However since the focus group participants primarily guided the discussions, this may have not been an issue and the use of the same group of content experts to assess the transcripts helped standardize the assessment.

Another question to consider is whether the focus group participants were representative of the population of interest. If the participants were not representative of their practice group, the themes elucidated from their responses may be incomplete or not applicable and therefore not valid for generalization, even in their practice group. There may have been a bias in that those who responded to the email invitation may have been more interested in pediatric HTN than their other coworkers. Therefore an unseen incentive may have been present from the beginning of the focus group sessions. This is a common problem with focus group work and this confounding effect was minimized by soliciting members of each group by group email, thereby not excluding any willing participants.

### Future plans

Based on these themes, multiple educational methods to address pediatric HTN recognition, evaluation and management are being formulated. We are developing multiple methods because we feel that there is no one approach that would address the majority of the themes. The goal of these educational methods would be to impart meaningful change on the part of the learner(s) so they may increase upon their continuous professional development by developing tools to become practitioners who are now agents of “transformative learning” [35]. As detailed by Kolb in the Kolb’s learning style construct, individuals may prefer any of four basic learning modes and it is important that methods to develop practitioner knowledge and skills provide opportunities for experiential learning that is robust and enduring. The challenge is to develop teaching and learning methods that promote this meaningful experiential learning so that the PCP must not only assimilate the medical knowledge (pediatric hypertension) but also become effective practitioners who can actively care for pediatric patients with hypertension [36].

To promote this active learning and skill development we have developed a short and useable BP algorithm for approaching pediatric essential HTN that can be used by pediatricians for learning to provide care in the clinical and office settings. Based on the focus group themes, we are also developing an online interactive module that will include the BP algorithm and incorporate other key points into an interactive Powerpoint® presentation by using an interactive software called Articulate®. This software transforms a Powerpoint® presentation into an engaging and interactive educational experience to reinforce key teaching points. These online modules will soon be made available to interested practitioners and appropriate outcomes assessed.

To provide another approach that serves those who prefer in-person education, we will also employ educational sessions in small group settings to reinforce key content and guidelines [37]. Self-learning materials will also be used that contain the same content. Pre and post tests will be used to determine a change in knowledge [38,39]. The true test will be to see if these educational methods change diagnostic accuracy, practice patterns with patients and referral patterns to our pediatric nephrology clinic [40-44]. If our primary care pediatricians become more comfortable recognizing, diagnosing and treating pediatric essential HTN, then referral rates of incorrectly identified patients with HTN should decline. More thoroughly evaluated and/or treated patients with HTN should increase among patient referrals to our pediatric nephrology clinic [45-47].

## Conclusions

In order to accurately recognize and diagnose at risk pediatric patients with HTN, practitioners must first be able correctly identify these patients, evaluate and manage them based on their BP readings. Busy practicing pediatricians are at the frontline of this task. Therefore they must be able to recognize patients with HTN or suspicion of HTN in an effective and appropriate manner and also be able to more effectively evaluate and/or treat children and adolescents with HTN based on their resources, experiences and interest. With the information gathered from these focus groups, we are committed to develop and implement effective educational methods to aid these practitioners and will provide methods to improve the care of children and adolescents with HTN in our region and beyond.

## Abbreviations

HTN: Hypertension; BP: Blood pressure; TLC: Therapeutic lifestyle changes; NCH: Nationwide Children’s Hospital; EHR: Electronic Health Record; PCP: Primary Care Pediatricians.

## Competing interests

The authors declare that they have no competing interests.

## Authors’ contributions

SC conceived of the study and was involved in each step the of the research process and was the main contributor with drafting the manuscript. He served as one of the expert transcript reviewers. DC provided the qualitative research design background and was instrumental in designing the study. JDM was the senior author who mentored SC and molded the research process and manuscript. He served as one of the expert transcript reviewers. All authors read and approved the final manuscript.

## Pre-publication history

The pre-publication history for this paper can be accessed here:

http://www.biomedcentral.com/1472-6920/14/154/prepub

## References

[B1] Din-Dzietham R Liu Y Bielo MV Sharma F High blood pressure trends in children and adolescents in national surveys, 1963 to 2002 Circulation 2007 116 13 1488 1496 17846287 10.1161/CIRCULATIONAHA.106.683243

[B2] Gomes RS Gomes RS Quirino IG Pereira RM Vitor BM Leite AF Oliveira EA Silva AC S e Primary versus secondary hypertension in children followed up at an outpatient tertiary unit Pediatr Nephrol 2011 26 3 441 447 21174218 10.1007/s00467-010-1712-x

[B3] Muntner P He J Cutler JA Wildman RP Whelton PK Trends in blood pressure among children and adolescents JAMA 2004 291 17 2107 2113 15126439 10.1001/jama.291.17.2107

[B4] The fourth report on the diagnosis, evaluation, and treatment of high blood pressure in children and adolescents Pediatrics 2004 114 2 Suppl 4th Report 555 576 15286277

[B5] Flynn J Zhang Y Solar-Yohay S Shi V Clinical and demographic characteristics of children with hypertension Hypertension 2012 60 4 1047 1054 22892814 10.1161/HYPERTENSIONAHA.112.197525

[B6] Flynn JT Pediatric hypertension: recent trends and accomplishments, future challenges Am J Hypertens 2008 21 6 605 612 18437129 10.1038/ajh.2008.159

[B7] Flynn JT Pediatric hypertension update Curr Opin Nephrol Hypertens 2010 19 3 292 297 20164767 10.1097/MNH.0b013e3283373016

[B8] Flynn JT Alderman MH Characteristics of children with primary hypertension seen at a referral center Pediatr Nephrol 2005 20 7 961 966 15864653 10.1007/s00467-005-1855-3

[B9] Flynn JT Falkner BE Obesity hypertension in adolescents: epidemiology, evaluation, and management J Clin Hypertens (Greenwich) 2011 13 5 323 331 21545393 10.1111/j.1751-7176.2011.00452.x PMC8108776

[B10] Robinson RF Batisky DL Hayes JR Nahata MC Mahan JD Body mass index in primary and secondary pediatric hypertension Pediatr Nephrol 2004 19 12 1379 1384 15503182 10.1007/s00467-004-1588-8

[B11] Sorof J Daniels S Obesity hypertension in children: a problem of epidemic proportions Hypertension 2002 40 4 441 447 12364344 10.1161/01.hyp.0000032940.33466.12

[B12] Manolio TA Olson J Longstreth WT Hypertension and cognitive function: pathophysiologic effects of hypertension on the brain Curr Hypertens Rep 2003 5 3 255 261 12724059 10.1007/s11906-003-0029-6

[B13] Croix B Feig DI Childhood hypertension is not a silent disease Pediatr Nephrol 2006 21 4 527 532 16491419 10.1007/s00467-006-0013-x

[B14] Falkner B Hypertension in children and adolescents: epidemiology and natural history Pediatr Nephrol 2010 25 7 1219 1224 19421783 10.1007/s00467-009-1200-3 PMC2874036

[B15] Collins RT II Alpert BS Pre-hypertension and hypertension in pediatrics: don’t let the statistics hide the pathology J Pediatr 2009 155 2 165 169 19619748 10.1016/j.jpeds.2009.02.006

[B16] Ilyas M Ellis EN Management of childhood hypertension: a guide for primary care physicians J Ark Med Soc 2006 103 6 137 140 17190413

[B17] Lieberman E Essential hypertension in children and youth: a pediatric perspective J Pediatr 1974 85 1 1 11 4604441 10.1016/s0022-3476(74)80276-3

[B18] Raj M Krishnakumar R Hypertension in children and adolescents: epidemiology and pathogenesis Indian J Pediatr 2012 80 1 S71 S76 22941155 10.1007/s12098-012-0851-4

[B19] Hansen ML Gunn PW Kaelber DC Underdiagnosis of hypertension in children and adolescents JAMA 2007 298 8 874 879 17712071 10.1001/jama.298.8.874

[B20] Lee AL Gordon AE Shaughnessy AF What should physicians know about hypertension? The implicit knowledge requirements in the maintenance of certification self-assessment module Fam Med 2007 39 4 280 283 17401773

[B21] Sacks FM Svetkey LP Vollmer WM Appel LJ Bray GA Harsha D Obarzanek E Conlin PR Miller ER 3rd Simons-Morton DG Karanja N Lin PH DASH-Sodium Collaborative Research Group Effects on blood pressure of reduced dietary sodium and the Dietary Approaches to Stop Hypertension (DASH) diet. DASH-Sodium Collaborative Research Group N Engl J Med 2001 344 1 3 10 11136953 10.1056/NEJM200101043440101

[B22] Morgan DL Focus Groups as Qualitative Research 1996 Thousand Oaks, CA: Sage

[B23] Maxwell J Qualitative Research Design 1996 Thousand Oaks, CA: Sage

[B24] Crabtree F Miller W Doing Qualitative Research 1999 Thousand Oaks, CA: Sage

[B25] Miles M Huberman AM Qualitative Data Anlaysis 1994 Thousand Oaks, CA: Sage

[B26] Glaser B Strauss AL The Discovery of Grounded Theory: Strategies for Qualitative Research 1967 New York: Aldine de Gruter

[B27] Strauss A Corbin J Basics of Qualitative Research: Techniques and Procedures for Developing Grounded Theory 1998 Thousand Oaks, CA: Sage

[B28] Constas M Qualitative analysis as a public event: the documentation of category development procedures Am Educ Res J 1992 29 254 266

[B29] Qualitative Research Review Guidelines http://www.biomedcentral.com/authors/rats

[B30] Podoll A Grenier M Croix B Feig DI Inaccuracy in pediatric outpatient blood pressure measurement Pediatrics 2007 119 3 e538 e543 17332173 10.1542/peds.2006-1686

[B31] Kalarchian MA Levine MD Arslanian SA Ewing LJ Houck PR Cheng Y Ringham RM Sheets CA Marcus MD Family-based treatment of severe pediatric obesity: randomized, controlled trial Pediatrics 2009 124 4 1060 1068 19786444 10.1542/peds.2008-3727 PMC2935494

[B32] Yoon EY Cohn L Rocchini A Kershaw D Freed G Ascione F Clark S Antihypertensive prescribing patterns for adolescents with primary hypertension Pediatrics 2012 129 1 e1 e8 22144698 10.1542/peds.2011-0877 PMC3255467

[B33] Varda NM Gregoric A A diagnostic approach for the child with hypertension Pediatr Nephrol 2005 20 4 499 506 15723196 10.1007/s00467-004-1737-0

[B34] Montgomery AA Fahey T A systematic review of the use of computers in the management of hypertension J Epidemiol Community Health 1998 52 8 520 525 9876364 10.1136/jech.52.8.520 PMC1756753

[B35] Armstrong EG Barsion SJ Creating “Innovator’s DNA” in Health Care Education Acad Med 2013 88 3 343 348 23348085 10.1097/ACM.0b013e318280cb7b

[B36] Kolb A Kolb DA Kolb’s Learning Styles Encyclopedia of the Sciences of Learning 2012 Berlin: Springer US 1698 1703

[B37] Sisson SD Rastegar D Rice TN Prokopowicz G Hughes MT Physician familiarity with diagnosis and management of hypertension according to JNC 7 guidelines J Clin Hypertens (Greenwich) 2006 8 5 344 350 16687943 10.1111/j.1524-6175.2006.05335.x PMC8109691

[B38] Drexel C Merlo K Basile JN Watkins B Whitfield B Katz JM Pine B Sullivan T Highly interactive multi-session programs impact physician behavior on hypertension management: outcomes of a new CME model J Clin Hypertens (Greenwich) 2011 13 2 97 105 21272197 10.1111/j.1751-7176.2010.00399.x PMC8673395

[B39] De Rivas B Barrios V Redón J Calderón A Effectiveness of an Interventional Program to Improve Blood Pressure Control in Hypertensive Patients at High Risk for Developing Heart Failure: HEROIC study J Clin Hypertens (Greenwich) 2010 12 5 335 344 20546374 10.1111/j.1751-7176.2010.00271.x PMC8673143

[B40] Dolor RJ Yancy WS Jr Owen WF Matchar DB Samsa GP Pollak KI Lin PH Ard JD Prempeh M McGuire HL Batch BC Fan W Svetkey LP Hypertension Improvement Project (HIP): study protocol and implementation challenges Trials 2009 10 13 19245692 10.1186/1745-6215-10-13 PMC2654882

[B41] Martinez-Valverde S Castro-Ríos A Pérez-Cuevas R Klunder-Klunder M Salinas-Escudero G Reyes-Morales H Effectiveness of a medical education intervention to treat hypertension in primary care J Eval Clin Pract 2012 18 2 420 425 21114796 10.1111/j.1365-2753.2010.01595.x

[B42] Campbell NR Tu K Brant R Duong-Hua M McAlister FA Canadian Hypertension Education Program Outcomes Research Task Force The impact of the Canadian Hypertension Education Program on antihypertensive prescribing trends Hypertension 2006 47 1 22 28 16344380 10.1161/01.HYP.0000196269.98463.fd

[B43] Tu K Davis D Can we alter physician behavior by educational methods? Lessons learned from studies of the management and follow-up of hypertension J Contin Educ Health Prof 2002 22 1 11 22 12004636 10.1002/chp.1340220103

[B44] Svetkey LP Pollak KI Yancy WS Jr Dolor RJ Batch BC Samsa G Matchar DB Lin PH Hypertension improvement project: randomized trial of quality improvement for physicians and lifestyle modification for patients Hypertension 2009 54 6 1226 1233 19920081 10.1161/HYPERTENSIONAHA.109.134874 PMC2784648

[B45] Bosworth HB Olsen MK Dudley T Orr M Goldstein MK Datta SK McCant F Gentry P Simel DL Oddone EZ Patient education and provider decision support to control blood pressure in primary care: a cluster randomized trial Am Heart J 2009 157 3 450 456 19249414 10.1016/j.ahj.2008.11.003

[B46] Herbert CP Wright JM Maclure M Wakefield J Dormuth C Brett-MacLean P Legare J Premi J Better Prescribing Project: a randomized controlled trial of the impact of case-based educational modules and personal prescribing feedback on prescribing for hypertension in primary care Fam Pract 2004 21 5 575 581 15367481 10.1093/fampra/cmh515

[B47] Cranney M Barton S Walley T Addressing barriers to change: an RCT of practice-based education to improve the management of hypertension in the elderly Br J Gen Pract 1999 49 444 522 526 10621984 PMC1313469

